# In Vitro Leishmanicidal Activity of Copaiba Oil and Kojic Acid Combination on the Protozoan *Leishmania (Leishmania) amazonensis* and Host Cell

**DOI:** 10.3390/microorganisms11122925

**Published:** 2023-12-05

**Authors:** Lienne Silveira de Moraes, Adan Jesús Galué-Parra, Amanda Anastácia Pinto Hage, Hévila Aragão Moura, Marcus Savio Araujo Garcia, Caroline Gomes Macêdo, Ana Paula Drummond Rodrigues, Giselle Maria Skelding Pinheiro Guilhon, Edilene Oliveira da Silva

**Affiliations:** 1 Pharmaceutical Sciences Post Graduation Program, Health and Biological Sciences Department, Federal University of Amapa (UNIFAP), Macapa 68903-419, AP, Brazil; liennemoraes@gmail.com; 2 Laboratory of Structural Biology, Institute of Biological Sciences, Federal University of Pará, Belém 66075-110, PA, Brazil; adangalue@gmail.com (A.J.G.-P.); amandahage@gmail.com (A.A.P.H.); hevilaragao@gmail.com (H.A.M.); garciamarcus28@gmail.com (M.S.A.G.); carolgmacedo123@gmail.com (C.G.M.); 3 National Institute of Science and Technology in Structural Biology and Bioimaging, Rio de Janeiro 21040-900, RJ, Brazil; anarodrigues@iec.gov.br; 4 Laboratory of Electron Microscopy, Evandro Chagas’s Institute, Department of Health Surveillance, Ministry of Health, Belém 70723-040, PA, Brazil; 5 Institute of Exact and Natural Sciences, Federal University of Pará, Belém 66075-110, PA, Brazil; giselle@ufpa.br

**Keywords:** copaiba oil, kojic acid, combination, tegumentary leishmaniasis, *Leishmania (Leishmania) amazonensis*

## Abstract

(1) Background: Leishmaniasis refers to a group of anthropozoonotic diseases caused by Leishmania. The major chemotherapeutic agent used for its treatment is Glucantime^®®^, but the search continues for new compounds that are economically viable and act on the protozoan without causing damage to the host cell. As an alternative approach, this study used a combination of copaiba oil (CO) and kojic acid (KA) to determine their in vitro action on host cells, on the *Leishmania (Leishmania) amazonensis* protozoan and its interaction with macrophages. (2) Methods: In vitro culture, analysis of cytokine release and microscopy assays were performed. Statistical analysis was performed with ANOVA (GraphPad Prism). (3) Results: The combination did not induce cytotoxic effects on macrophages after treatment but promoted morphological changes in the protozoan, such as nuclear alterations (apoptotic characteristics), alterations in the cellular body and an increase in the number of electrodense structures and acidocalcisomes, observed mainly at the concentrations of CO20KA50 and CO30KA50 μg/mL. We observed reductions in the intracellular amastigote number and in the production of proinflammatory cytokines, such as IL-6 and TNF-α, after treatment with CO30KA at 50 µg/mL. (4) Conclusions: We report here, for the first time, that the combination of CO and KA may be a promising approach against *Leishmania (Leishmania) amazonensis*.

## 1. Introduction

Leishmaniasis is a complex disease that is considered a serious public health problem, constituting a set of polymorphic characteristics and caused by protozoan species belonging to the genus Leishmania, with more than 12 million infected people and 350 million people at risk of infection [1,2]. Leishmaniasis is considered one of the six major tropical diseases in the world, being endemic in 98 countries, with most cases reported in tropical and subtropical regions, including Latin American countries and the African and Asian continents [2,3,4]. Each year, an estimated 2 million people are infected by the parasite, leading to approximately 50,000 deaths/year [2,4]. In Brazil, leishmaniasis has an endemic character, with the north and northeast regions of the country registering the highest number of cases of the disease for more than thirty years [3]. Leishmaniasis is transmitted by sandflies of the *Lutzomyia* genera (New World) and *Phlebotomus* (Old World), which become amastigote forms after the infective metacyclic promastigote forms invade the host cells (macrophages), thereby establishing infection [5,6].

Macrophages are central cells in the immune response to infection by protozoa of the genus Leishmania and are activated by helper T lymphocytes because of the production of Interferon-γ (IFN-γ), allowing the system to eliminate the parasite due to the production of reactive oxygen species (ROS) and nitric oxide (NO). Alternatively, these cells can be silenced by the parasite by evasion mechanisms that are triggered, thus allowing their survival and successful infection [5,6,7,8,9]. The treatment used for leishmaniasis has been the same since the 1940s, with pentavalent antimonials (Sb^+5^) as the drugs of choice [2,3,10]. As second-line drugs for the treatment of American Tegumentary Leishmaniasis (ATL), pentamidine and amphotericin B are used in patients who do not respond or have any antimony restriction [11,12].

Studies have shown that some factors limit and make it difficult to treat leishmaniasis with existing drugs, such as strain resistance, the presence of several clinical forms, high cost, high toxicity, adverse reactions and treatment invasiveness [13,14]. Leishmaniasis is a disease with limited treatment; therefore, the search continues for alternative substances for the treatment of the disease, offering different modes of action and that are low-cost, less invasive and less toxic. Among these substances, bioproducts are highlighted in the search for these new products, mainly in the Amazon region, which presents rich biodiversity [15].

Previous studies have demonstrated that different substances and bioproducts have leishmanicidal activity [16,17]. Kojic acid (KA), a natural product produced by *Aspergillus*, *Penicillium* fungi and *Acetobacter* [18,19], is a promising substance against leishmaniasis because it is a potent activator of macrophages [20] and has been previously described as a leishmanicidal agent [21]. Additionally, copaiba oil (CO), extracted from *Copaifera* sp., presented action on promastigote and amastigote forms of the protozoan *Leishmania (L.) amazonensis* [22]. 

Considering the ability of KA to promote the activation of murine macrophages in addition to its leishmanicidal properties in vitro and the leishmanicidal action of CO [23], we hypothesize that combinations of compounds are able to improve the leishmanicial effects of both substances. Therefore, the aim of this study was to evaluate the action of the combination of these two bioproducts on the host cell and on the protozoan, *Leishmania (L.) amazonensis*, the agent causing ATL. Furthermore, an important implication of the study would be the preparation of a product for topical use for the treatment of cutaneous leishmaniasis.

## 2. Materials and Methods

### 2.1. Obtaining and Dilution of the Copaíba Oil and Kojic Acid

Resin oil number 7 (tree identification code) was extracted from the species *Copaifera reticulata* DUCKE, collected in the Tapajós National Forest, Pará, Brazil (presented as major constituents, trans-α-bergamotene (30.3%) and β-bisabolene (26%)), according to Herrero-Ja’uregui et al., 2011 [23]. The oil was provided by Dra. Giselle Guilhon, from the Chemistry Institute, Federal University of Pará and Kojic Acid (KA) was obtained from Sigma Aldrich^®®^. KA was diluted in a culture medium, and copaiba oil was solubilized in DMSO (10%) and diluted in a culture medium, with a stock concentration of 1 mg/mL. KA was used at a concentration of 50 µg/mL, according to Rodrigues et al., 2014 [21] and CO was used at concentrations of 10, 20 and 30 µg/mL, after a previous viability experiment, according to Santos et al., 2011 [22].

### 2.2. Tests with the Host Cell

#### 2.2.1. Peritoneal Macrophage Culture

Macrophages were obtained from the peritoneal cavity of male BALB/c mice (*Mus musculus*), and the animals were sacrificed in a CO_2_ chamber (Insight^®®^). The material was harvested with Hank’s solution, concentrated via centrifugation at 4 °C, cultured in a 24-well plate and incubated in an atmosphere containing 5% CO_2_ at 35 °C for 1 h. After, nonadherent cells were washed with DMEM and incubated for 24 h with DMEM supplemented with 10% fetal bovine serum (FBS). The animals were euthanized in accordance with the norms of the Ethics Committee (Commission of ethics in animal research—Evandro Chagas’s Institute, Certified n° 23/2015) and packed in plastic containers suitable for disposal in selective collection containers for biological material and discarded according to ANVISA RDC306/04. 

#### 2.2.2. Macrophage Treatment and Cytotoxic Analysis

The cytotoxicity procedure was performed according to Fotakis and Timbrell [24] with some modifications. Macrophages were treated with different combinations of CO and KA (CO10AK50, CO20AK50, CO30AK50, CO50AK50, CO100AK50, CO200AK50, CO400AK50, CO600AK50 and CO800AK50 µg/mL) for 72 h in a humidified atmosphere containing 5% CO_2_ at 37 °C. After, the cells were incubated with MTT (0.5 mg/mL) dissolved in phosphate-buffered saline (PBS), pH 7.2, for 3 h, dimethylsulfoxide (DMSO) was added to the wells, and the plate was allowed to shake for 10 min for complete solubilization. The absorbance of the solutions was recorded at an optical density (OD) of 570 nm using a spectrophotometer (Bio-Rad Model 450 Microplate Reader). Assay specificity was determined using nonviable cells treated with 10% formaldehyde. The results are expressed as the optical density.

### 2.3. Macrophage Microbicidal Response after Treatment

#### 2.3.1. Detection of Reactive Oxygen Species (ROS) Production

Macrophages were cultured and treated with three different combinations (CO10AK50, CO20AK50 and CO30AK50 µg/mL). After, the cells were washed with PBS pH 7.2 and incubated with the green fluorescent marker CellROX^®®^ (Molecular Probes Invitrogen) at a concentration of 5 μM diluted in DMEM culture medium as described by Ahn et al. [23], with some modifications. After 30 min in an atmosphere containing 5% CO_2_ at 37 °C, the cells were washed with PBS pH 7.2, and then the wells were scraped. The solution containing the cells was analysed using a flow cytometer (BD FACS Canto TM II), and the result was expressed as the mean fluorescence intensity (MFI). As a positive control, the cells were treated with 100 nM LPS and IFN-γ for 24 h, and as a negative control, the cells were not stained with CellROX^®®^.

#### 2.3.2. Indirect Nitric Oxide (NO) Production

The evaluation of nitrite/nitrate dosage in a culture medium is an indirect way to determine the NO concentration produced in macrophages. For this quantification, the Griess method was performed, consisting of the addition of 50 µL of Griess reagent (1% sulfanilamide in 5% phosphoric acid and 0.1% naphthylenediamine in distilled water) and 50 µL of cell supernatant. After the treatment period, the culture supernatant was removed for indirect NO measurement. The reading was recorded at an optical density (OD) at 570 nm using a spectrophotometer (BIO-RAD Model 450 Microplate Reader), and the nitrate concentration was expressed as the optical density.

### 2.4. Tests with Leishmania (Leishmania) amazonensis

#### 2.4.1. Cultivation and Maintenance

*Leishmania (L.) amazonensis* promastigotes (MHOM/BR/26361) were obtained in NNN (Neal–Novy–Nicolle) medium from Evandro Chagas’s Institute and subsequently maintained in RPMI medium supplemented with 10% heat-inactivated FBS at 27 °C and kept in a BOD incubator at 27 °C.

#### 2.4.2. Antipromastigote Assay

*Leishmania* (L.) *amazonensis * promastigotes were added to the culture wells (1 × 10^6^ parasites/mL) and treated with different combinations of CO and KA (CO10KA50, CO20KA50, CO30KA50, CO50KA50, CO100KA50, CO200KA50, CO400KA50, CO600KA50 and CO800KA50 µg/mL) for 72 h. Then, 100 μL of the culture was removed and incubated with MTT (2 mg/mL) for 4 h, and 20 μL of DMSO was added to completely solubilize the crystals for 30 min with agitation. The resulting solution was recorded at an optical density (OD) of 570 nm using a spectrophotometer (BIO-RAD Model 450 Microplate Reader). As a positive control, amphotericin-B (AMB) was used at a concentration of 0.5 μg/mL.

#### 2.4.3. Microscopy Analysis

Treated and untreated (control group) promastigote cultures were processed for light microscopy (LM), scanning electron microscopy (SEM) and transmission electron microscopy (TEM). For LM, cells were fixed with 4% formaldehyde, adhered to coverslips containing poly-L-lysine for 30 min, stained with Giemsa for 30 min at room temperature and analysed under an Axio Scope A1-Zeiss microscope. For SEM, promastigotes were fixed with 4% formaldehyde and 2% glutaraldehyde in 0.1 M cacodylate buffer, pH 7.2, for 1 h, were adhered to coverslips containing poly-L-lysine, postfixed in 1% osmium tetroxide, dehydrated in ethanol, critical point dried (CO_2_ in air), coated with gold and examined with an SEM–LEO 1450VP. For TEM, promastigotes were fixed with 2.5% glutaraldehyde and 4% freshly prepared formaldehyde in 1x PHEM buffer (MgCl_2_-5 mM; KCl-70 mM; EGTA-10 mM; HEPES-20 mM; PIPES-60 mM), pH 7.2, for 1 h at room temperature. Cells were washed and postfixed in a solution containing 1% osmium tetroxide, 0.8% ferrocyanide and 5 mM calcium chloride for 1 h. Subsequently, the cells were washed, dehydrated in graded acetone and embedded in Epon^®®^ resin. Ultrathin sections were obtained in a Leica EM UC6 ultramicrotome and stained with uranyl acetate and lead citrate. Sections were examined using a Zeiss EM900 TEM. The positive control AMB (0.5 µg/mL) was used. 

#### 2.4.4. ROS Detection

After treatment with three different combinations (CO10AK50, CO20AK50 and CO30AK50 µg/mL), a CellROX^®®^ Green kit (Molecular Probes Invitrogen, Carlsbad, CA, USA) was used to detect ROS at a concentration of 7.5 μM. After 45 min of incubation at 27 °C, promastigotes were washed with PBS pH 7.2, and cells were analysed on a BD FACSCantoIITM flow cytometer with BD FACSDiva software, 8.0 version.

#### 2.4.5. Apoptosis/Necrosis Detection by Flow Cytometry

Promastigotes were treated as described above, and then, the cells were removed from the culture, centrifuged, and washed with PBS pH 7.2 for incubation for 30 min with 10 μL of Annexin V-FITC, incubated with 10 μL of PI for 10 min, and read in a BD FACSCantoIITM flow cytometer. Data were analysed using FlowWin, software, 2.5.1 version (Turku, Finland) and GraphPad Prism 8.0 version. AMB (0.5 µg/mL) was used as a positive control to induce necrosis, and miltefosine (3 µg/mL) was used as a positive control for apoptosis.

### 2.5. Interaction: Host Cell and Protozoan

#### 2.5.1. Antiamastigote Assay

Peritoneal macrophages were infected with *Leishmania (L.) amazonensis* promastigotes at a proportion of 10:1 (parasites:macrophages) for 4 h at 35 °C in an atmosphere containing 5% CO_2_. After incubation, washes were performed to remove noninternalized parasites, and cells were treated for 72 h with the three different combinations (CO10KA50, CO20KA50 and CO30KA50) under the same conditions mentioned above. Cells were fixed with 4% formaldehyde, stained with Giemsa and mounted on glass slides containing Entellan^®®^ (Merck^®®^, Darmstadt, Germany). To determine the number of parasites within the macrophages of each group, 100 cells were counted per coverslip, and the percent inhibition was determined compared to the control group (100% survival) and combinations. As a positive control, Glucantime^®®^ was used at a concentration of 50 μg/mL, according to Rodrigues et al. [21].

#### 2.5.2. Indirect Nitric Oxide (NO) Production

The culture supernatant of the interaction was removed for NO measurement using the Griess method, according to Moraes et al. [14]. The resulting solution was recorded at an optical density (OD) of 570 nm using a spectrophotometer (BIO-RAD Model 450 Microplate Reader).

#### 2.5.3. Cytokine Profile Analysis

The supernatant of macrophages infected with *Leishmania (L.) amazonensis* was used to quantify pro- and anti-inflammatory cytokine profiles using a cytometric bead assay (CBA Th1/h2/Th17 mouse—IL-2, IL4, IL6, IL10, IL17A, INF-γ and TNF-α) according to the manufacturer’s instructions. The samples were analysed using a BD FACSCantoII flow cytometer using FACSDiva software, 8.0 version (BD Biosciences, Carlsbad, CA, USA), and the analysis was performed in FCAP Array 3.0 and GraphPad Prism 5.0. The results are expressed as pg/mL and were calculated according to a standard curve.

### 2.6. Statistical Analysis

All experiments were performed in triplicate. The mean and standard deviation of three experiments were determined. Statistical analysis was performed using one-way ANOVA, followed by the Tukey test, performed using GraphPad Prism 5.0 (GraphPad Software, La Jolla, CA, USA.). A *p* value < 0.05 was considered statistically significant.

## 3. Results

### 3.1. Macrophage Viability following Combination Treatment

**Figure 1:**
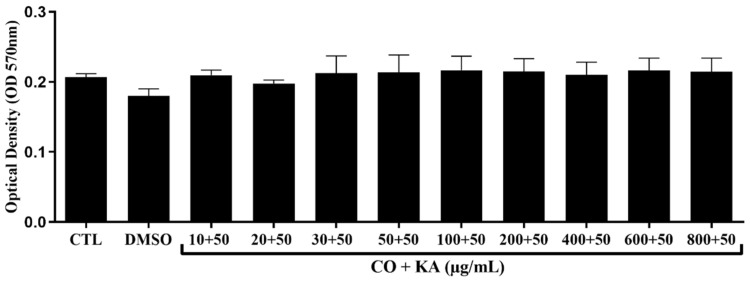
Cell viability using the MTT method of peritoneal macrophages maintained in culture for 72 h and treated with different concentrations of CO combined with 50 µg/mL KA . The data showed that there was no reduction in mitochondrial metabolism when compared to the control group without treatment. Data represent the mean of three independent experiments, followed by ANOVA (analysis of variance) and Tukey’s test, with *p* < 0.05. CTL: control group; DMSO: dimethylsulfoxide; CO+KA: Copaiba oil + kojic acid.

Macrophage viability was measured using the MTT assay (Figure 1), and after analysis, the combination of CO and KA at the concentrations tested did not show a cytotoxic effect on the macrophages after treatment when compared to the control group without treatment (Figure 1). CO did not show cytotoxic effect towards macrophage (Figure S1).

### 3.2. Microbicide Response of Macrophages

**Figure 2:**
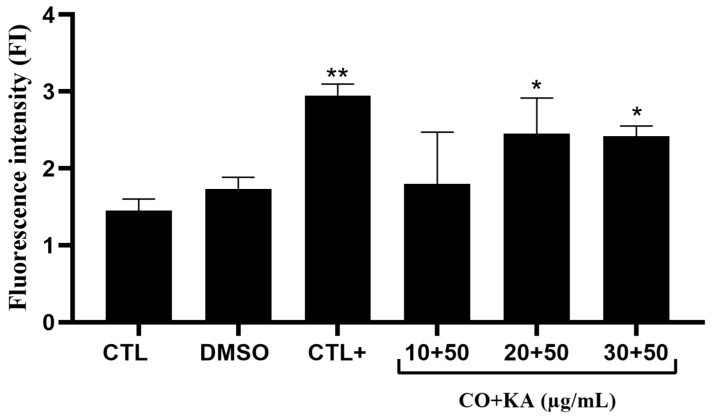
Reactive oxygen species (ROS) production detection in macrophages treated with CO and KA using CellROX^®®^ dye. Note the increase in ROS production in the CO20KA50 and CO30KA50 μg/mL groups compared to untreated cells (CTL). Data represent the mean of three independent experiments, followed by ANOVA (analysis of variance) and Tukey’s test. (*) *p* < 0.05., (**) *p*<0.01 CTL: control group; DMSO: dimethylsulfoxide; CTL+: 50 nM LPS (Lipopolysaccharide); CO+KA: Copaiba oil + kojic acid.

After 72 h of treatment, significant ROS production in macrophages treated with CO20KA50 µg/mL and CO30KA50 µg/mL was observed compared to the control group without treatment. Lipopolysaccharide (LPS—50 nM) was also used as a positive control for ROS production in macrophages (Figure 2).

### 3.3. Morphological Analysis

**Figure 3:**
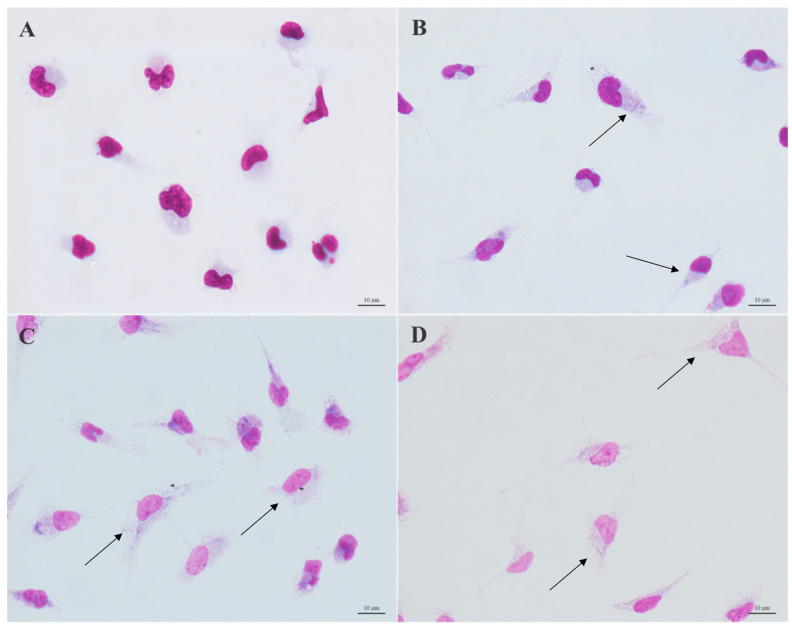
Morphological analysis of untreated and treated macrophages. (**A**) control group. (**B**) macrophages treated with combination CO10KA50 μg/mL, (**C**) treated with combination CO20KA50 μg/mL and (**D**) treated with combination CO30KA50 μg/mL. Note the apparent cell spreading, increase in the number of cytoplasmic projections (arrows) and number of vacuoles (*), compared to the untreated group. Scale bar: 10 µm.

Optical microscopy analysis showed that after 72 h of macrophage treatment, the combination of CO and KA promoted changes in the cells (Figure 3). Figure 3A shows macrophages from the control group, without treatment with the combination, with typical morphology. Macrophages treated with the combination (CO10KA50, CO20KA50 and CO30KA50 μg/mL) showed more apparent cell activation, an increase in the number of cytoplasmic projections (arrows) and an increase in the number of vacuoles (*), compared to the control group (Figure 3B–D).

### 3.4. Combination Promoted Antipromastigote Activity and Reactive Oxygen Species Production, Significant Morphological Alterations and Cell Death by Initial Apoptosis

**Figure 4:**
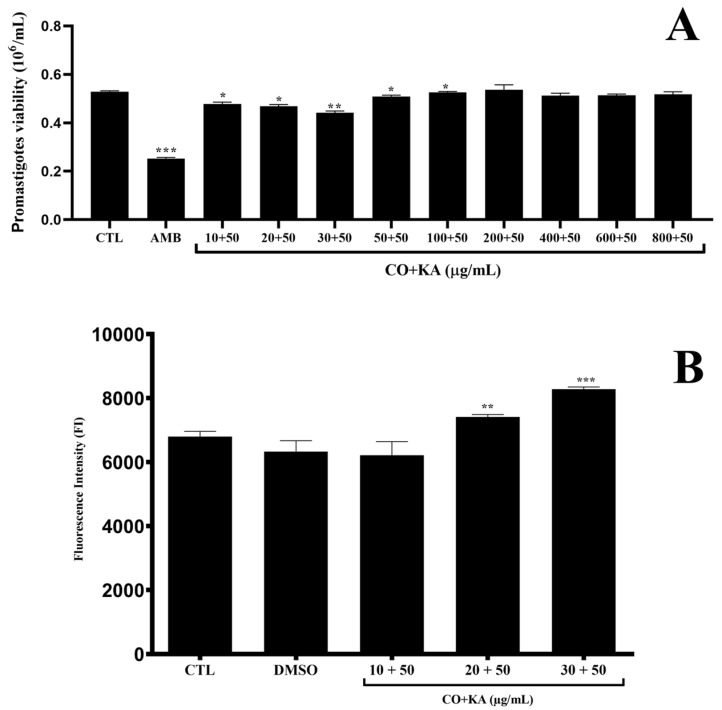
(**A**) Cell viability of the promastigote forms of *Leishmania (L.) amazonensis* maintained in culture for 72 h and treated with different combinations of CO and KA (CO10KA50, CO20KA50 and CO30KA50 µg/mL) by MTT assay. Note the reduction in viability at lower CO concentrations combined with KA. (**B**) ROS production analysis with CellROX dye in *Leishmania (L.) amazonensis* promastigotes treated with three combinations (CO10KA50, CO20KA50 and CO30KA50 µg/mL) for 72 h. Note the increased production of reactive oxygen species at concentrations of 20 and 30 µg/mL of CO combined with 50 µg/mL of KA. Data represent the mean of three independent experiments, followed by ANOVA (analysis of variance) and Tukey’s test, with (*) *p* <0.05, (**) *p* < 0.01 and (***) *p* < 0.001. CTL: control group; DMSO: dimethylsulfoxide; CO+KA: Copaiba oil + kojic acid.

Treatment with CO and KA promoted a reduction in the viability of promastigote forms when compared to the untreated group, indicating that these associations have leishmanicidal activity in this evolutive form of the parasite (Figure 4A). As a positive control, AMB (0.5 μg/mL) was used and promoted a significant reduction in promastigote form viability (Figure 4A). CO promoted slight reduction in viability of promastigote form when tested alone (Figure S2). Combinations of CO20KA50 and CO30KA50 µg/mL promoted a significant increase in the production of ROS in promastigotes compared to the control group (Figure 4B).

**Figure 5:**
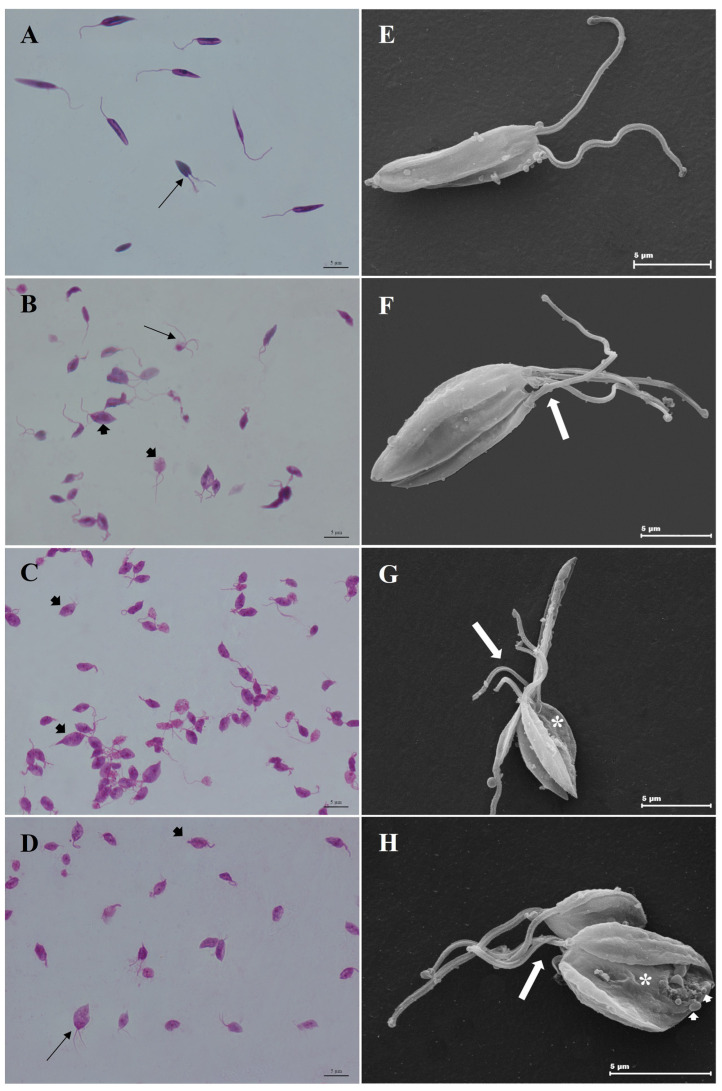
Morphological alterations in promastigote forms after combination treatments. (**A**–**D**) Light microscopy. (**A**) Untreated promastigotes. Typical morphology of promastigotes, with elongated cell body, single flagellum, and typical cell division process (arrow); (**B**) promastigotes treated with CO10KA50 μg/mL; (**C**) parasites treated with CO20KA50 μg/mL; (**D**) treated with CO30KA50 μg/mL. Parasites with more than one flagellum emerging from the flagellar pocket (arrow) and round or ovoid bodies (black arrowhead). Scale bar, 5 µm. (**E**–**H**) Scanning electron microscopy (SEM). (**E**) Untreated promastigotes, with typical morphology, evidencing promastigotes in division; (**F**) parasites treated with CO10KA50 μg/mL; (**G**) treated with CO20KA50 μg/mL; (**H**) treated promastigotes with CO30KA50 μg/mL. Note promastigotes with more than one flagellum emerging from the flagellar pocket (white arrow), cell body retraction, surface changes (*) and the presence of structures at the back of the cell body called “blebs” (white arrowhead).

Analysis using LM showed alterations in the promastigote forms (stationary phase) after treatment with the combinations CO10KA50, CO20KA50 and CO30KA50 µg/mL. We observed a shortening of the cell body and the presence of more than one flagellum emerging from the flagellar pocket, demonstrating atypical cell division (Figure 5B–D) when compared to the control group (5A) that did not receive treatment, presenting typical morphology. SEM analysis showed promastigotes with typical morphology, an elongated cell body and a single flagellum emerging from the flagellar pocket (Figure 5E). In promastigotes treated with combinations, we observed shortening of the cell body, demonstrating a rounded appearance (*), significant changes in the cell membrane (*), promastigotes with atypical division, with more than two flagellum emerging from the flagellar pocket (white arrow), in combinations CO10KA50 and CO20KA50 µg/mL (Figure 5G and Figure 5H, respectively) and protuberances distributed in the plasma membrane of the parasite (blebs—white arrowhead) in the combination CO30KA50 µg/mL (Figure 5H) when compared to the untreated group (Figure 5E). 

**Figure 6:**
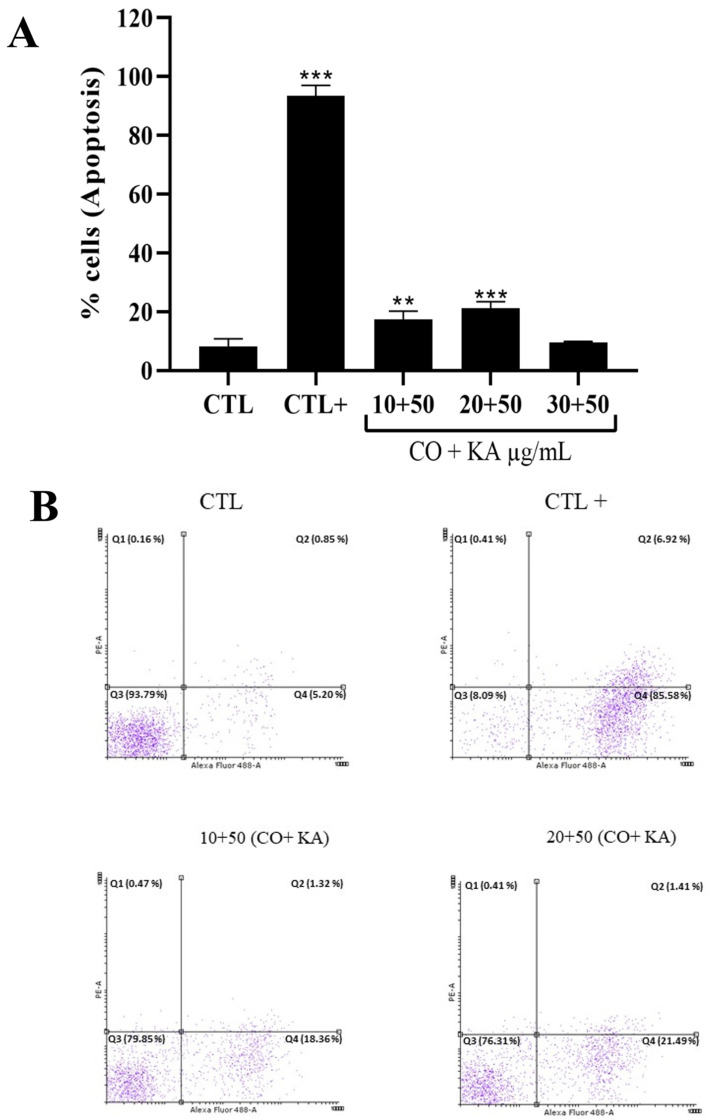
Labelling Annexin-V and PI in promastigotes of *Leishmania (L.) amazonensis* after 72 h of incubation with the CO+KA combination. (**A**) Percentage of parasites labelled with Annexin V; a reduction in the percentage of labelled cells was observed in the presence of CO10KA50 and CO20KA50 µg/mL compared with the untreated promastigotes (control group—CTL), indicating that these parasites are in the initial process of apoptosis. (**B**) Dot plot representative of the labelling with PI and annexin-V in the CTL group, untreated cells and CTL^+^ (treated cells with miltefosine at 3 μg/mL for 24 h), treated cells with miltefosine at 3 μg/mL for 24 h and parasites treated with CO10KA50 and CO20KA50 µg/mL. In the images, quadrant Q_1_ represents cells labelled with PI (PI^+^/Annexin V^−^), Q_2_ represents cells with double labelling or double positive group (PI^+^/Annexin V^+^), characterizing cells in late apoptosis, Q_3_ represents unlabelled cells or double negative group (PI^-^/Annexin V^−^), and Q_4_ represents parasites labelled only with Annexin V (PI^-^/Annexin V^+^), characterizing cells in the initial process of apoptosis. Data represent the mean of three independent experiments, followed by ANOVA (analysis of variance) and Tukey’s test, with (**) *p* < 0.01 and (***) *p* < 0.001. CTL: control group; CO+KA: Copaiba oil + kojic acid.

Flow cytometry analysis of the promastigotes labelled with propidium iodide (PI) and Annexin-V FITC showed that there was an increase in the percentage of cells positively labelled for Annexin-V in the combinations of CO10KA50 and CO20KA50 µg/mL compared to the untreated promastigotes and positive control, promastigotes treated with miltefosine (3 µg/mL), showing that these cells are in the initial process of apoptosis (Figure 6A,B). There was no increase in positively labelled cells for Annexin-V and PI, which characterizes a late apoptosis process, or only with PI, which characterizes cells in the necrosis process (Figure 6A).

### 3.5. Combination of CO and KA Promoted Ultrastructural Alterations in Promastigotes

**Figure 7:**
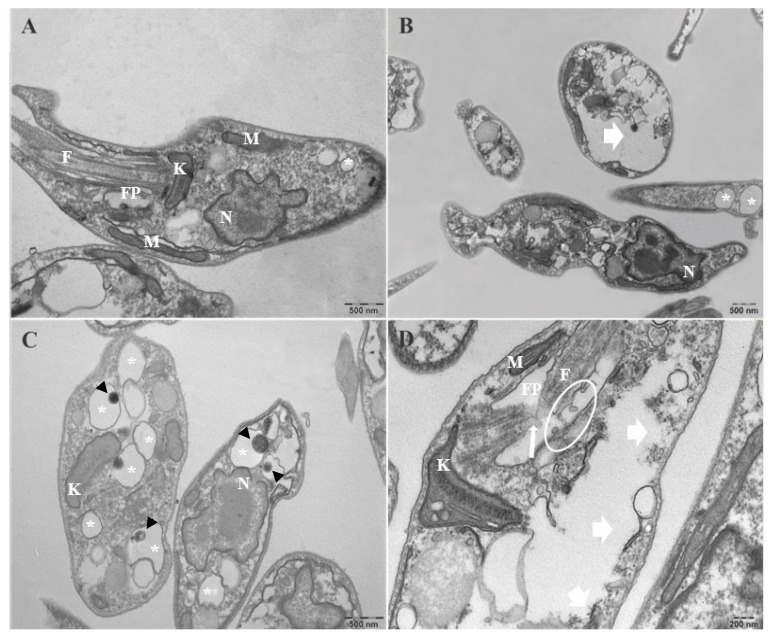
Electromicrography of promastigotes of *Leishmania (L.) amazonensis* treated with CO+KA for 72 h showed ultrastructural changes, as observed via TEM. (**A**) Control group: untreated promastigotes. Note the typical morphology. (**B**) Promastigotes after the addition of CO10KA50 μg/mL. (**C**) Promastigotes after the addition of CO20KA50 μg/mL. (**D**) Promastigotes after addition of CO30KA50 μg/mL. Disruption of the flagellum (white arrow) and cell body (white arrow) membrane was observed. F—flagellum; N—nucleus; K—kinetoplast; M—mitochondria; FP—flagellar pocket; (*) acidocalcisomes, electron-dense material (black arrowhead).

Ultrastructural analysis through the use of TEM allowed us to observe the changes caused by the addition of the combination at different concentrations in promastigote forms. Control group promastigotes exhibited typical morphology without changes in the plasma membrane, a characteristic nucleus, a kinetoplast and mitochondrion with typical morphology and a flagellar pocket with only one flagellum (Figure 7A). Combination treatment promoted modifications in the nucleus, with morphology suggestive of apoptosis, including chromatin condensation, cell body disruption (white arrowhead) (Figure 7B,C), apparent increase in the number of membrane-bound structures, acidocalcissomes (*), electron-dense material inside (black arrowhead) (Figure 7C), kinetoplast swelling, cell body disruption (white arrowhead), flagellar membrane disruption (white arrow) and increase in the number of vesicles in the flagellar pocket (Figure 7D) when compared to the control group (Figure 7A).

### 3.6. Combination Promotes Reduction in Amastigote Forms of Protozoan Leishmania (L.) amazonensis, after Interaction

**Figure 8:**
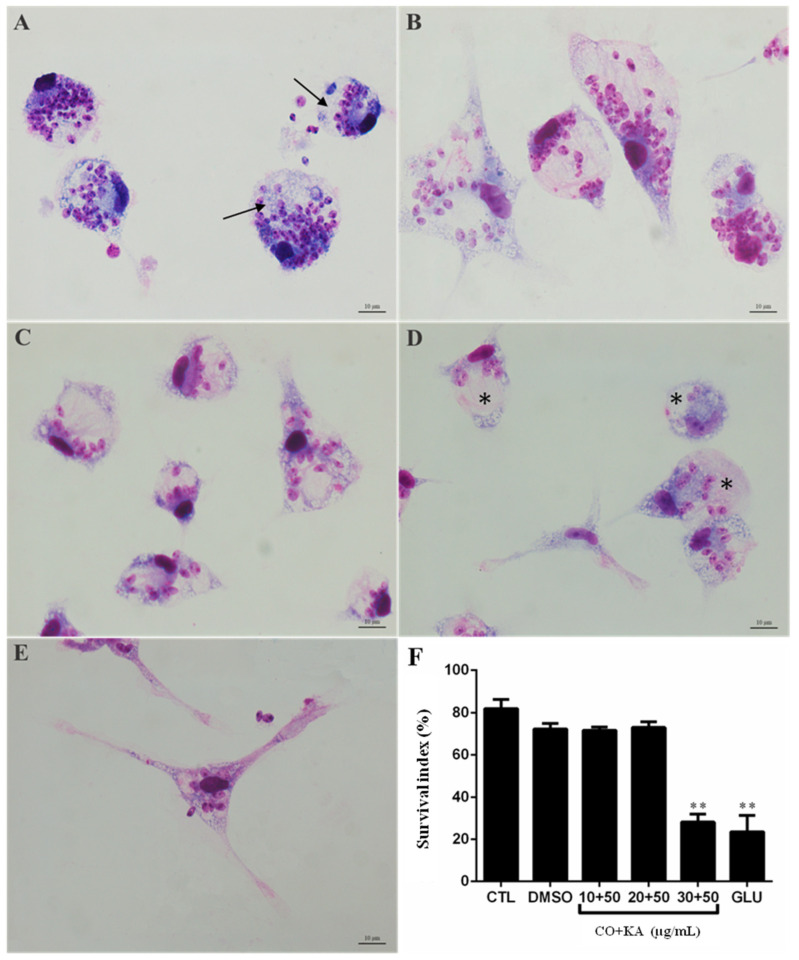
Effect of the compounds on the intracellular forms of the protozoan, *Leishmania (L.) amazonensis*. (**A**) CTL cells without treatment, parasites can be observed inside the host cell (arrows); (**B**) cells treated with the CO10KA50 µg/mL combination; (**C**) cells treated with CO20KA50 µg/mL; (**D**) cells treated with CO30KA50 µg/mL. Note the reduction in the number of amastigotes ( asterisk); (**E**) cells treated with glucantime (50 µg/mL); (**F**) quantification of amastigote forms. Data represent the mean of three independent experiments, followed by ANOVA (analysis of variance) and Tukey test, with (*) *p* <0.05 and (**) *p* < 0.01. CTL: control group (without treatment); CO+KA: Copaiba oil + Kojic acid.

To evaluate the antiamastigote activity of the CO+KA combination, macrophages infected with *Leishmania (L.) amazonensis* were treated for 72 h. A decrease of 77,5% in the number of amastigotes after treatment with CO30KA50 μg/mL (Figure 8F) was observed compared to the untreated group (CTL). Glucantime^®®^ at a concentration of 50 μg/mL (Figure 8E) was used as the reference drug according to Rodrigues et al. [21]. LM showed that after treatment with the combination, macrophages were more spread out, with large vacuoles (*), cytoplasmic projections, and few amastigotes inside (Figure 8D) when compared with infected and untreated macrophages (Figure 8B–D).

### 3.7. Immunomodulatory Effect on Infected Macrophages

**Figure 9:**
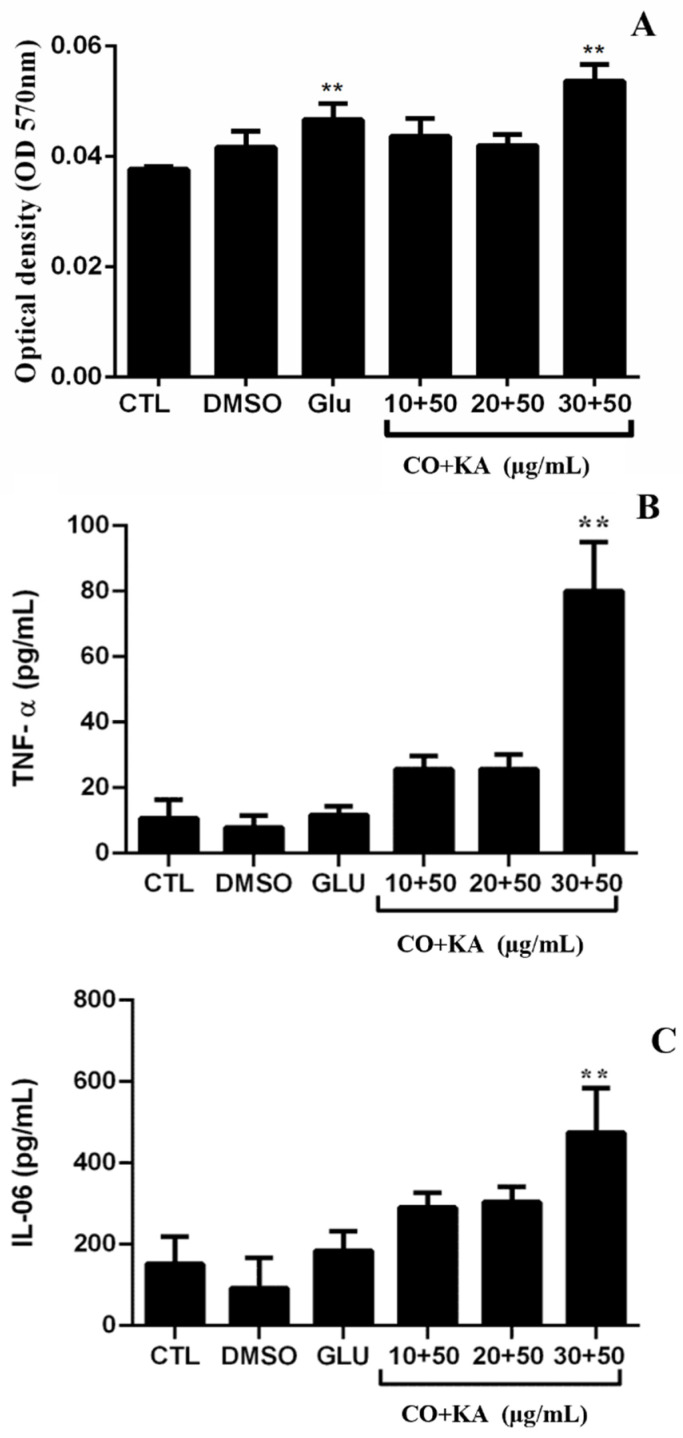
NO production and cytokine profiles in macrophages infected with *Leishmania (L.) amazonensis* and treated with the combination for 72 h. (**A**) Increase in indirect NO production only with CO30KA50 μg/mL. (**B**,**C**), cytokine production in the supernatant of macrophages infected with *Leishmania (L.) amazonensis* and treated. (**B**) TNF-α quantification and (**C**) IL-6 quantification, only in CO30KA50 μg/mL. Data represent the mean of three independent experiments, followed by ANOVA (analysis of variance) and Tukey’s test, with (**) *p* < 0.01. CTL: control group; CO+KA: Copaiba oil + kojic acid.

To verify whether the compounds induced NO production in macrophages after infection with *Leishmania (L.) amazonensis*, nitrite/nitrate was measured, with an increase in indirect NO production only with CO30KA50 μg/mL when compared to the untreated cells (Figure 9A). The cytokine profile was evaluated in the culture supernatant of macrophages infected with *Leishmania (L.) amazonensis* and treated with the combination using flow cytometry. The results demonstrated a significant increase in the cytokines TNF-α (Figure 9B) and IL-6 (Figure 9C) in the CO30KA50 μg/mL group compared to the untreated group.

## 4. Discussion

The treatment for leishmaniasis, although effective, has some limitations and disadvantages, such as high toxicity, need for long periods of treatment, invasive administration, high cost and resistance of some strains of the parasite [25,26,27]. The search for new drugs from natural sources with leishmanicidal activity has attracted the attention of researchers [27]. Some bioproducts described in the literature have broad biological activity, such as anti-inflammatory, antitumour, antimicrobial, antifungal, antimelanogenic activities as well as activity against some pathogens, especially against protozoa. Macrophages (host cells) have an important role in the immune response, as they have potent antimicrobial functions [21,28,29,30,31].

The present study demonstrated the action of copaiba oil (CO) combined with kojic acid (KA), demonstrating the action and possible effects of this combination in macrophages, promastigote and amastigote forms of protozoan *Leishmania (L.) amazonensis*. 

Evaluating the action of these substances in the protozoan and in the host cell (macrophages) is needed because several substances are present in their composition, making cytotoxic action important in this cell type. Oils from more than 20 species of Copaifera are used in folk medicine in Brazil, and significant differences in chemical composition occur among them [32]. Here, we report on Copaifera reticulata DUCKE, characterized by its major constituents, trans-α-bergamotene (30.3%) and β-bisabolene (26%), which differ from those of other species [23].

Macrophages are central cells of the immune response, play a role in the recognition of pathogenic microorganisms and are important during infection with protozoa of the genus *Leishmania*, as the species *Leishmania (L.) amazonensis* can escape the microbicidal response and survive inside cells [33]. Therefore, the search for new substances capable of activating the macrophage response without affecting the viability and survival of the parasite is important.

The combination of CO with KA, as shown through the MTT method, did not affect the mitochondrial metabolism of macrophages, an important finding in the search for an alternative therapy for treatment. These results confirm the data observed by Rodrigues et al. [20]. The authors showed that the isolated kojic acid did not affect the viability of peritoneal macrophages, and Santos et al. [22], who studied copaiba oil extracted from the species *Copaifera reticulata*, demonstrated no cytotoxic action on macrophages of the J774.G8 strain. Other studies have also demonstrated that resin oil extracted from the species *Copaifera reticulata* did not alter the viability of peritoneal macrophages until the concentration of 500 µg/mL [32]. In a previous study, Soares et al. [34] demonstrated that the resin oil of different species of *Copaifera* sp. was not cytotoxic for murine macrophages, corroborating the findings in the present study.

Treatment with the combination (CO+KA) induced activation of macrophages, as observed through the increase in cell spreading and cytoplasmic projections, as well as by the significant increase in ROS production. The production of microbicidal substances, such as ROS by the cell characterizes the so-called “oxidative burst”, which makes the cellular environment hostile to parasite survival, helping in its destruction [35]. These data agree with a study by Rodrigues et al. [20], which demonstrated that KA treatment could promote changes in the morphology of the host cell as well as activation through the production of superoxide radicals. Changes in the morphology of these cells caused by treatment with the combination of bioproducts, such as increased adhesion, cell spreading and cytoplasmic projections, are important, as such changes characterize the cell activation process, which favors the cell’s resistance against parasites intracellularly.

Tests with the protozoan are important when searching for new therapeutic targets for the treatment of leishmaniasis. The viability test (MTT) in promastigotes of *Leishmania (L.) amazonensis*, incubated with the combination (CO+KA), after 72 h demonstrated a reduction in the viability of these cells using CO5AK50 to CO100AK50 µg/mL. Our data are similar to those of Rodrigues et al. [21] when they used KA and Santos et al. [22] when they used CO, which showed a reduction in cell viability after incubation with the bioproducts. 

The synergy between two compounds does not always lead to death, but it can trigger important mechanisms that lead to impaired cell multiplication and development. Even with these characteristics, more studies with the combination of the bioproducts are necessary to establish the mechanisms of action on the cell and on the protozoan [36].

LM analysis of promastigote forms treated with the combination (COKA) showed important alterations, such as shortening of the cell body and the presence of more than one flagellum emerging from the flagellar pocket. SEM analysis confirmed the changes observed via LM, such as shortening of the cell body, presence of more than one flagellum emerging from the flagellar pocket, in addition to irregularity on the surface of the promastigotes and increase in protuberant structures called *blebs*. *Blebs* are structures that can be formed by various mechanisms and have different functions, such as demonstrating that the cell is undergoing atypical division or apoptosis [37].

Ultrastructural analysis via TEM demonstrated that treatment with the combination promoted important changes in the morphology of the *Leishmania (L.) amazonensis* promastigote, including the presence of nuclei with apoptotic characteristics after treatment with CO20AK50 µg/mL, increase in acidocalcisomes with electron-dense content inside and swelling of the kinetoplast after treatment with CO20KA50 and CO30KA50 µg/mL, and cell body and flagellar membrane disruption and increase in the number of vesicles in the flagellar pocket after treatment with CO30KA50 µg/mL.

Acidocalcisomes are membrane-bound organelles that are involved in the stock of calcium and other ions that are important for the parasite’s survival [38]. These organelles are involved in the response to stress suffered by the parasite during its cell cycle, either by changing the pH of the medium or by treatment with certain drugs [39]. Because this organelle is important for parasite survival, treatment with the combination of bioproducts seems to increase this structure. Further studies are needed to determine whether the structures altered are acidocalcissomes and the possible mechanism of action.

Another important organelle for the parasite is the kinetoplast, which is responsible for the synthesis and storage of a DNA molecule. The swelling process caused by treatment with bioproducts can interfere with the synthesis of this molecule and, consequently, with the development of the parasite.

Similar results were demonstrated by Santos et al. [22,40], who used only oil extracted from the *C. reticulata* species and showed parasites exhibiting both kinetoplast and membrane alterations. In addition, the authors demonstrated that there were changes in the cell division process, such as changes in the cell body and organelles of the *Leishmania (L.) amazonensis* promastigotes. The treated promastigote forms had a rounded shape with the presence of more than two flagella emerging from the flagellar pouch. These findings corroborate our study, and changes in the morphology of promastigote forms may affect important processes for parasite survival, such as recognition, cell adhesion, growth regulation, and the expression of surface antigens and receptors [41].

In the present study, the combination of bioproducts could induce a significant increase in the production of ROS in promastigotes treated with CO30KA50 μg/mL. Calixto et al. [42] demonstrated that quinolone QDS3 increases the production of ROS in *Leishmania (L.) amazonensis* promastigotes, suggesting an action on the mitochondria that can cause damage to the parasite. Increased levels of ROS can induce oxidative stress, causing changes in both the shape and important protozoan organelles, as well as damage to nucleic acids and proteins [43,44].

To confirm the presence of nuclei with apoptotic characteristics observed through TEM, labelling with Annexin-V was performed. Treatment with CO20KA50 µg/mL indicated an initial process of cell death by apoptosis, corroborating what was observed when using ultrastructural analysis.

Other studies have shown that bioproducts induce cell death of the parasite via apoptosis. Da Silva et al. [45] demonstrated that the aqueous extract of *Physalis angulata* promoted cell death via apoptosis in the species *Leishmania infantum*. The apoptosis mechanism in protozoan Leishmania promastigotes can be induced by some drugs used to treat leishmaniasis, such as liposomal amphotericin B [46].

Leishmania is an obligatory intracellular parasite of which the host cells are part of the mononuclear phagocytic system. The search for new compounds that can act on these cells without causing damage to the host cells and that help to destroy the parasitic amastigote form is important. Thus, in the present work, the treatment of infected macrophages was performed to determine whether the combination of bioproducts would have an effect on the intracellular (amastigote) form of the protozoan *Leishmania (L.) amazonensis*. The results showed that treatment for 72 h with CO30KA50 µg/mL promoted a reduction of 77.5% in the number of amastigotes inside the vacuole when compared to the control group without treatment. The combination action was more effective on amastigotes, likely due to the direct action that the treatment caused on the host cell and indirect on the parasite. Kian et al. [47] demonstrated that treatment with copaiba oil extracted from the species *Copaifera martii* could reduce the proliferation of amastigotes of *Trypanosoma cruzi*. Another study that corroborates our findings was presented by Rodrigues et al. [21]. These authors showed that in vitro treatment with KA could reduce the number of amastigotes of the protozoan *Leishmania (L.) amazonensis* inside the macrophages.

The infected and treated cells could produce NO, which is likely related to CO, as KA does not induce the production of NO by macrophages [20,48]. This result is notable as *L. (L.) amazonensis* can inhibit the microbicidal response of the host cell [49], and treatment with the combination could reverse this effect.

As macrophages are part of the immune response and actively participate in the interaction with the parasite, showing the immunomodulatory effect of these bioproducts was interesting. Analysis of the cytokine profile produced by macrophages infected with *L. (L.) amazonensis* and treated with the combination of CO and KA made it possible to observe that treatment with the combination of bioproducts stimulated demonstrated the production of the proinflammatory cytokines TNF-α and IL-6. As previously described, the cytokine TNF-α is involved in the cell activation process through Toll-like receptors, and IL-6 is mainly associated with the development and multiplication of Th17 cells [50,51].

Thus, the combination of CO and KA could promote the modulation of the response of infected macrophages, leading to a reduction in amastigote forms within these cells. The changes promoted by the combination indicate a profile of activation of this cell by the M1 pathway (classical pathway), as the proinflammatory cytokines IL-6 and TNF-α and increased NO were produced, in addition to the morphological changes caused by combination treatment, such as cell spreading and increased cytoplasmic projections observed through optical microscopy analysis. These characteristics that demonstrate the M1 activation profile have been described by several authors, corroborating the findings of the present study [52,53]. Da Costa et al. [48] observed that treatment with 50 µg/mL KA promoted an increase in IL-6 in human monocytes. Another important finding that corroborates our findings was demonstrated by Santiago et al. [54], who observed an increase in the production of TNF-α by human monocytes after treatment with *Copaifera* sp. oil-resin.

The investigation of natural products that present great potential for the pharmaceutical industry in the discovery of new agents that can act to combat neglected diseases is of great importance today. Some products extracted from plants have biological activity attributed to components existing in their formulations, which belong to several groups, such as alkaloids, terpenes, quinones, and isoquinolones [16,22,55]. Many of these natural components have previously been identified as potential agents to treat leishmaniasis, and more research can contribute to the discovery of products that are more effective against this disease.

Therefore, the results of the present study showed that treatment with CO+KA could promote activation of the host cell by increasing cell spreading and ROS and NO production, which was also important to promote the reduction in intracellular amastigote forms, likely through the macrophage microbicidal mechanism. Therefore, the combination of bioproducts may represent an alternative for the topical treatment of American cutaneous leishmaniasis. However, this current approach, using treated infected macrophages in vitro, has limitations that need to be overcome using pre-clinical animal models to ensure the therapeutic potential of these combined natural products.

## 5. Conclusions

Cutaneous leishmaniasis (CL) is a serious public health problem in developing countries. Current treatments are expensive, require parenteral administration and cause serious side effects. Combinations of products isolated from natural sources could be a therapeutic alternative for leishmaniasis treatment. Thus, the present study demonstrates that the use of Copaiba oil and Kojic Acid combined presented important effects against *Leishmania (Leishmania) amazonensis* parasites. In vitro culture, analysis of cytokine release and microscopy assays were performed in this study. The combination promoted morphological changes in the promastigote forms, induced an initial process of cell death (apoptosis) and reduced the number of amastigotes within the macrophage. Additionally, the combination also promoted the activation of host cells through cell spreading and the production of ROS without changing the viability of these cells. It also stimulated a microbicidal response in protozoan-infected macrophages through the production of NO and proinflammatory cytokines. Therefore, the use of natural therapy is of great importance for native populations in low-income countries that use medicinal plants to treat many neglected diseases, such as CL. We hope that this research holds promise for the preparation of a combined natural product for topical use as an alternative treatment for CL.

## Data Availability

The data presented in this study are available on request from the corresponding author.

## Supplementary Materials

The following supporting information can be downloaded at: https://www.mdpi.com/article/10.3390/microorganisms11122925/s1, Figure S1. Effect of different concentrations of copaiba oil on peritoneal macrophage viability for 72 h. Figure S2: Effect of different concentrations of copaiba oil on promastigotes of *Leishmania (Leishmania) amazonensis* for 72 h.
