# Supplementary material for: In Vitro Leishmanicidal Activity of Copaiba Oil and Kojic Acid Combination on the Protozoan Leishmania (Leishmania) amazonensis and Host Cell
Source: Microorganisms. 2023 Dec 5;11(12):2925. doi: 10.3390/microorganisms11122925 (PMC10745933; doi:10.3390/microorganisms11122925)
Supplement: Supplementary file 1 [file microorganisms-11-02925-s001.zip › microorganisms-2592678-supplementary.pdf]

Supplementary Materials

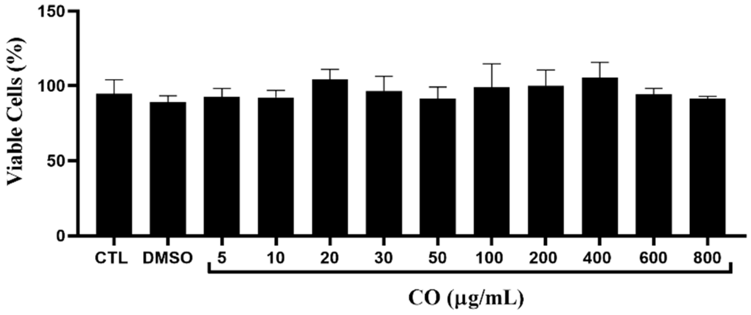

Figure S1. Effect of different concentrations of copaiba oil (CO) on peritoneal macrophage viability for 72 hours. CTL (Control), DMSO (Dimethyl sufoxide) was used as negative control.

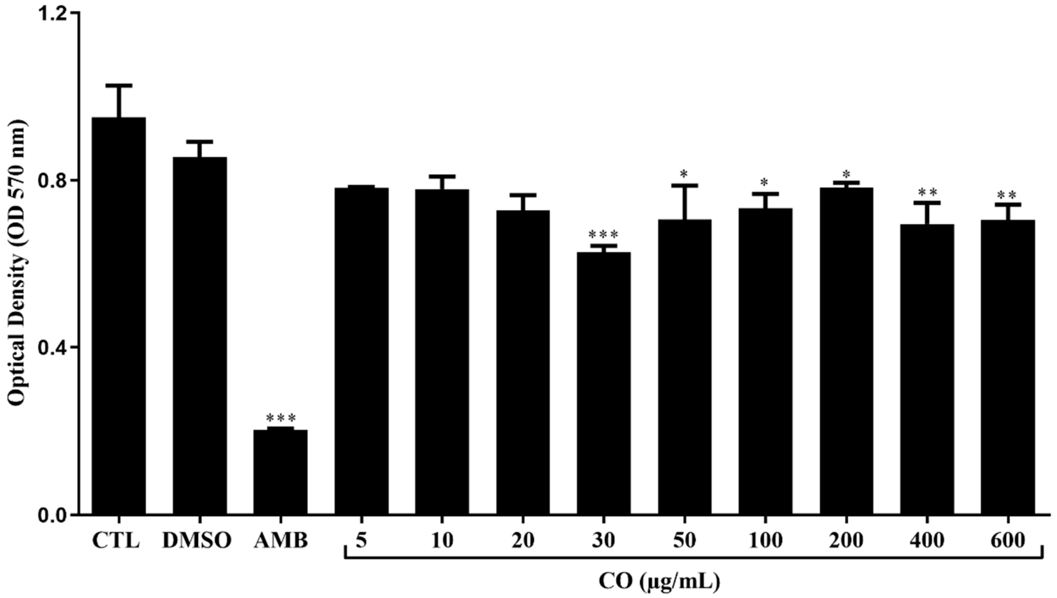

Figure S2. Effect of different concentrations of copaiba oil (CO) on promastigotes of *Leishmania (Leishmania) amazonensis* for 72 hours. CTL(Control),DMSO (Dimethyl Sufoxide) was used as negative control, ANF-B (Amphotericin-B) was used as positive control. *p<0.05, ** p<0.01, ***p<0.001
